# Field evaluation of an immunochromatographic test for diagnosis of cystic and alveolar echinococcosis

**DOI:** 10.1186/s13071-018-2896-3

**Published:** 2018-05-23

**Authors:** Chun-hua Gao, Jun-yun Wang, Feng Shi, Dietmar Steverding, Xia Wang, Yue-tao Yang, Xiao-Nong Zhou

**Affiliations:** 1 National Institute of Parasitic Diseases, Chinese Center for Disease Control and Prevention, WHO Collaborating Centre for Tropical Diseases, National Center for International Research on Tropical Diseases, Ministry of Science and Technology, Key Laboratory of Parasite and Vector Biology, Ministry of Health, Shanghai, China; 2 Bob Champion Research & Education Building, Norwich Medical School, University of East Anglia, Norwich, UK

**Keywords:** Cystic echinococcosis, Alveolar echinococcosis, *Echinococcus granulosus*, *Echinococcus multilocularis*, Abdominal ultrasound image examination, Rapid diagnostic test

## Abstract

**Background:**

The larval stages of the tapeworms *Echinocoocus granulosus* and *Echinococcus multilocularis* are the causative agents of human cystic echinococcosis (CE) and human alveolar echinococcosis (AE), respectively. Both CE and AE are chronic diseases characterised by long asymptomatic periods of many years. However, early diagnosis of the disease is important if treatment and management of echinococcosis patients are to be successful.

**Methods:**

A previously developed rapid diagnostic test (RDT) for the differential detection of CE and AE was evaluated under field conditions with finger prick blood samples taken from 1502 people living in the Ganzi Tibetan Autonomous Prefecture, China, a region with a high prevalence for both forms of human echinococcosis. The results were compared with simultaneously obtained abdominal ultrasonographic scans of the individuals.

**Results:**

Using the ultrasonography as the gold standard, sensitivity and specificity, and the diagnostic accuracy of the RDT were determined to be greater than 94% for both CE and AE. For CE cases, high detection rates (95.6–98.8%) were found with patients having active cysts while lower detection rates (40.0–68.8%) were obtained with patients having transient or inactive cysts. In contrast, detection rates in AE patients were independent of the lesion type. The positive likelihood ratio of the RDT for CE and AE was greater than 20 and thus fairly high, indicating that a patient with a positive test result has a high probability of having echinococcosis.

**Conclusions:**

The results suggest that our previously developed RDT is suitable as a screening tool for the early detection of human echinococcosis in endemic areas.

## Background

Human echinococcosis is one of the world’s most serious parasitic zoonosis caused by larval stages of cestodes belonging to the genus *Echinococcus* (family Taeniidae). Among them, cystic echinococcosis (CE) caused by *E. granulosus* and alveolar echinococcosis (AE) caused by *E. multilocularis* are the most common forms of the disease [1, 2]. Both CE and AE mainly affect the liver, causing chronic disease. Human echinococcosis is not only a serious medical condition but also a social and economic problem for vulnerable populations [3]. CE has a worldwide distribution whereas AE occurs mainly in the northern hemisphere in higher latitudes. A further complication is that the two forms of human echinococcosis are co-endemic in many areas of the world [4–8].

Currently, diagnosis of human echinococcosis is based predominantly on imaging techniques. Ultrasound is the commonly used method for diagnosis of both CE and AE. Using ultrasound, the location, number and size of cysts or lesions can be identified, although in a time-consuming way. However, small-sized lesions may not be detected by ultrasonography and the technique is not particularly suitable for the diagnosis of pulmonary echinococcosis as the air in the lung and the sternum interfere with the imaging. Different traditional serological tests based on antibody detection are widely used for the diagnosis of human echinococcosis to complement imaging-based examination, especially when imaging features are unclear [9–13]. However, imaging and traditional immunological tests require well-equipped facilities and well-trained staff.

Rapid diagnostic tests (RDTs) such as immunochromatographic tests are extremely easy to apply; they are rapid, field-portable and do not need special laboratory equipment, and therefore, are particularly useful in resource-limited settings. Several reports have been published describing the performance of commercial and experimental RDTs for the diagnosis of echinococcosis [14–18]. With the exception of the dot immunogold filtration assay (DIGFA) developed by Feng et al. [17], these RDTs cannot be used for differential diagnose of CE and AE. We have developed an immunochromatographic tests for simultaneous diagnosis of AE and CE that showed excellent diagnostic performance in laboratory evaluation [19]. This RDT device has been recently certificated by the Chinese FDA and is commercially available.

The aim of the present work was to evaluate the effectiveness of our previously developed RDT for the detection of CE and AE under field conditions. As study site, the Ganzi Tibetan Autonomous Prefecture was chosen because the eastern Tibetan plateau has the highest prevalence of human echinococcosis worldwide and both CE and AE are co-endemic in this region [20–25].

## Methods

### Study area

The study was conducted in Ganzi Tibetan Autonomous Prefecture (27°58' to 34°20'N, 97°22' to 102°29'E) from May to October in 2016. The region is situated in the west of the Sichuan Province on the Tibetan Plateau. More than three-quarters (78.4%) of the population in the area are Tibetans.

### Study subjects

Outpatients with clinical suspicion of echinococcosis (abdominal pain or discomfort, poor appetite, marasmus, etc.) were presented voluntarily at the local hospital or at the Centre for Disease Control in the Ganzi Prefecture. Patients who previously had surgery for echinococcosis or who were under medication for echinococcosis were excluded from the study.

### Ultrasonography and immunodiagnostic test

Ultrasound examinations and immunodiagnostic tests were carried out by different members of staff at the same time and their results were blinded to each other. Before initiation of the study, a training course in ultrasound examination and in the use of the RDT device, and in interpretation and recording of results was provided.

Study participants were given an abdominal ultrasonographic scan using a portable ultrasound machine (GE LOGIQ Book XP, General Electric, Boston, USA). The recorded ultrasound images were read by two experienced sonographers and graded as CE, AE, CE plus AE, other lesions or normal. The classification of CE cysts and AE lesions were based on stage-specific ultrasound images [26–28]. It should be noted that AE lesions were graded according to the US classification because it offers better value in field screenings as it provides information about lesion size.

The RDT device (cystic echinococcosis and/or alveolar echinococcosis antibody gold immunochromatographic assay test kit; Shanghai Xinjier Biotechnology Co. Ltd., Shanghai, China) used in this study was previously developed by our study group [19] and certificated by the Chinese FDA under the registration no. 20163400065. This RDT uses crude hydatid cyst fluid (HCF) and a recombinant 18 kDa protein (rEm18) as antigens for the detection of *E. granulosus* and *E. multilocularis* antibodies in serum samples, respectively [19]. Whole blood samples were collected from each participant by finger prick and 20 μl was immediately applied to the RDT device. Results were read after 15 min and recorded according the product’s instruction as follows: only control line turned pink = negative for CE and AE; control line and test line 1 (HCF antigen) turned pink = positive for CE; control line and test line 2 (rEM18 antigen) turned pink as well as control line, test line 1 (HCF antigen) and test line 2 (rEM18 antigen) turned pink = positive for AE. As for most AE cases as well as for CE/AE co-infections both test lines appear pink (i.e. the test does not discriminate between *E. multilocularis* and *E. granulosus/E. multilocularis* infections), AE single infections and CE/AE co-infections were confirmed by ultrasonography.

The results of ultrasound examinations and immunodiagnostic tests were eventually matched by an independent expert.

### Data analysis

Diagnostic parameters (sensitivity, specificity, positive and negative predictive values, positive and negative likelihood ratios, and accuracy) for the performance of the RDT were calculated from a 2 × 2 table using the ultrasound imaging results as a gold standard. The parameters were computed using the online diagnostic test evaluation calculator MEDCALC (https://www.medcalc.org/calc/diagnostic_test.php).

## Results

A total of 1502 people with suspicion of echinococcosis living in the Ganzi Tibetan Autonomous Prefecture were simultaneously examined by abdominal ultrasonography and tested for the presence of echinococcosis antibodies using our RDT device. Echinococcal cysts and lesions were found in 440 patients by ultrasonography while no evidence of an echinococcosis infection was detected in the remaining 1062 individuals. Of the echinococcosis patients, 275 were diagnosed with CE and 165 with AE (Table 1). Cysts and lesions of all echinococcosis cases were exclusively found in the liver. However, as only abdominal ultrasonography was performed, it remained unknown whether any of the apparently uninfected individuals had echinococcal cysts elsewhere in their bodies (e.g. lungs). No dual infection with both CE and AE was found in any patient. It should also be mentioned that none of the patients were found to be infected with other helminths or other pathogens.

**Table 1:** Results of ultrasound imaging and RDT of 1502 people from the Ganzi Prefecture

Cyst or lesion type	No. of confirmative ultrasound images	No. of confirmative RDT results		
		CE positive (%)	AE positive (%)	CE/AE negative (%)
CE	275	259 (94.2)	0 (0)	16 (6.8)
CE1	86	85 (98.8)	0 (0)	1 (1.1)
CE2	116	114 (98.3)	0 (0)	2 (1.7)
CE3a	45	43 (95.6)	0 (0)	2 (4.4)
CE3b	16	11 (68.8)	0 (0)	5 (31.2)
CE4	7	4 (57.1)	0 (0)	3 (42.9)
CE5	5	2 (40.0)	0 (0)	3 (60.0)
AE	165	0 (0)	161 (97.6)	4 (2.4)
AE1	7	0 (0)	6 (85.7)	1 (14.3)
AE2	70	0 (0)	68 (97.1)	2 (2.9)
AE3	49	0 (0)	48 (98.0)	1 (2.0)
AEf^a^	39	0 (0)	39 (100)	0 (0)
Negative	1062	31 (2.9)	45 (4.2)	986 (92.8)

^a^Includes AE2f and AE3f cases

According to the criteria for the classification of ultrasound images of echinocoocsis [26–28], about three-quarters (73.5%) of CE cases were determined to have CE1- or CE2-type cysts while about one-fifth (22.2%) had CE3-type cysts and only a few (~ 4%) had CE4- or CE5-type cysts (Table 1). Of the 275 CE cases, 259 tested positive for the presence of CE antibodies with our RDT (Table 1). Interestingly, considerably more patients with active CE1-, CE2- and CE3a-type cysts showed a positive reaction (95.6–98.8%) than patients with transient/inactive CE3b-, CE4- and CE5-type cysts (40.0–68.8%) (Table 1). For the patient group with active cysts, the overall positive percentage was 98.0% (242 out of 247) with a 95% confidence interval of 97.7–98.2%. For the patient group with transient/inactive cysts, the overall positive percentage was 60.7% (17 out of 28) with a 95% confidence interval of 57.1–69.7%. Two non-overlapping confidence intervals provide strong evidence that the proportion positive was indeed different in the two groups and not due to chance.

Of the 165 AE cases, the majority of patients (72%) had AE2- or AE3-type lesions (Table 1). About a quarter of patients (23.6%) had AE2f- and AE3f-type lesions that were grouped together as cases with AEf-type lesions (Table 1). Only a few cases (~ 4%) were determined to have AE1-type lesions (Table 1). Of the 165 patients with AE lesions, 161 tested positive for the presence of AE antibodies with our RDT (Table 1). In contrast to CE cases, for AE cases no obvious dependency was observed between lesion type and positive antibody response as most patients with any AE lesion showed a positive reaction (87.5–100%) (Table 1).

Of the 1062 individuals whose abdominal ultrasound was negative for the presence of echinococcosis cysts or lesions, between 3–4% gave a positive reaction with our RDT for the apparent presence of *E. granulosus* and/or *E. multilocularis* antibodies (Table 1). These patients are under observation for the development of echinococcosis.

Using the results of the ultrasound examination as a gold standard, the diagnostic parameters for the performance of our RDT under field conditions were calculated. Sensitivity and specificity of the RDT for the detection of both CE and AE were quite high (> 94%) (Table 2). The positive predictive values were significantly lower than the negative predictive values (especially for AE) (Table 2) indicating that the RDT in the current epidemiological situation of echinococcosis in the study area was somewhat better in correctly identifying negative results. However, the high positive likelihood ratios (> 20) and the low negative likelihood ratios (< 0.1) (Table 2) provide strong evidence that our RDT can be used to diagnose both CE and AE. The accuracy of our RDT was very similar for detection of both CE and AE (Table 2), and with values of ≥ 96%, the probability that patients were correctly identified was quite high.

**Table 2:** Diagnostic performance of RDT in the detection of CE and AE

Diagnostic parameter	CE (95% CI)	AE (95% CI)
True positive (tp)	258	161
True negative (tn)	1031^a^	1017^b^
False positive (fp)	31	45
False negative (fn)	16	4
Sensitivity^c^ (%)	94.2 (90.7–96.6)	97.5 (93.9–99.3)
Specificity^d^ (%)	97.1 (95.9–98.0)	95.8 (94.3–96.9)
Positive likelihood ratio^e^	32.3 (22.8–45.7)	23.0 (17.3–30.7)
Negative likelihood ratio^f^	0.06 (0.04–0.10)	0.03 (0.01–0.07)
Positive predictive value^g^ (%)	89.3 (85.5–92.2)	78.2 (72.8–82.7)
Negative predictive value^h^ (%)	98.5 (97.6–99.0)	99.6 (99.0–99.9)
Accuracy^i^ (%)	96.5 (95.3–97.4)	96.0 (94.8–97.0)

*Abbreviation*: *CI* confidence interval

^a^True negative = total no. of negative minus no. of CE positive (1062 minus 31)

^b^True negative = total no. of negative minus no. of AE positive (1062 minus 45)

^c^Sensitivity = $$ \frac{\mathrm{tp}}{\mathrm{tp}+\mathrm{fn}} $$ × 100

^d^Specificity = $$ \frac{\mathrm{tn}}{\mathrm{tn}+\mathrm{fp}} $$ × 100

^e^Positive likelihood ratio = $\frac{\mathrm{sensitivity}}{1-\mathrm{specificity}}$

^f^Negative likelihood ratio = $\frac{1-\mathrm{sensitivity}}{\mathrm{specificity}}$

^g^Positive predictive value = $\frac{\mathrm{tp}}{\mathrm{tp}+\mathrm{fp}}$

^h^Negative predictive value = $\frac{\mathrm{tn}}{\mathrm{tn}+\mathrm{fn}}$

^i^Accuracy = $$ \frac{\mathrm{tp}+\mathrm{tn}}{\mathrm{tp}+\mathrm{tn}+\mathrm{fp}+\mathrm{fn}} $$

## Discussion

Human echinococcosis is considered a neglected zoonotic disease [29] and can be categorised as an ‘infectious diseases of poverty’ [30]. Diagnosis of the disease is mainly based on ultrasound imaging examination and conventional immunological tests. In underserved rural endemic areas, there is usually a general lack of facilities and an absence of well-trained health-workers restricting the diagnosis of echinococcosis by imaging and serological techniques. An ideal diagnostic test should be sensitive, specific, suitable for the use in the field and easy to perform and interpret. RDTs such as immunochromatographic tests are usually extremely easy to apply. In addition, they are rapid, field-proven, inexpensive, and particularly useful in resource-poor settings. Several commercial and experimental RDTs for the diagnosis of CE or AE have been developed [14–16], but studies evaluating these devices under field conditions are very limited [17, 31]. Therefore, we previously developed an RDT which can detect simultaneously CE and AE with high sensitivity and specificity [19]. The results of the present study showed that our RDT can diagnose infections with *E. granulosus* and *E. multilocularis* in > 94% in patients with hepatic CE cysts and in > 97% with hepatic AE lesions, respectively, when tested under the current epidemiological situation of echinococcosis in the Ganzi region. It is worth noting that sensitivity, specificity and accuracy of our RDT determined under field conditions were comparable to those previously established under laboratory conditions [19].

Likelihood ratios are independent of the prevalence of a disease in a population and, therefore, are very useful measures for the diagnostic accuracy of a diagnostic test [32]. The high positive likelihood ratios of our RDT confirm that a patient with a positive test result has a high probability of being infected with *E. granulosus* or *E. multilocularis*. On the other hand, the low negative likelihood ratios of our RDT indicate a low risk of error when excluding an *Echinococcus* infection in individuals with a negative test result. These findings indicate that our RDT has fairly high sensitivity and accuracy in the detection of both CE and AE.

Although very high detection rates were found for patients with CE1-, CE2- and CE3a-type cysts, considerably lower detection rates were obtained with patients having CE3b-, CE4- and CE5-type cysts. This outcome may be due to the fact that the latter cyst types are transient (CE3b-type cyst) or inactive with calcifications (CE4- and CE5-type cysts) [26] releasing only minimal amounts of or even no antigen [33]. As a consequence, the patient’s immune response would decrease over time and lower amounts of antibodies would be produced after a while. In contrast, high detection rates were found for patients with any type of AE lesions. However, patients with AE1 lesions usually show weak immune responses [28]. The high seropositive rate in AE1 patients observed in this study may be due to their small number that might have biased the results.

Quite a few individuals (76 in total) with no ultrasound evidence of abdominal CE cysts or AE lesions gave a positive reaction with our RDT. However, the corresponding percentage proportions were relatively small in these groups (2.9 and 4.3% for apparent CE and AE detection, respectively) and close to the false positive rates previously determined for the test with healthy donors under laboratory conditions (3.3 and 1.7% for apparent CE and AE detection, respectively [19]). Another explanation could be that these positive-tested subjects had cysts or lesions that were too small to be detectable by the imaging technique used. This may also be an indication for the limitation of ultrasonography in the detection of echinococcosis during the early stages of the infection. Alternatively, these individuals may have had cysts or lesions in other organs, with the lungs being the second most primarily infected organ in about 11–14% of CE patients [34, 36] while primary extra-hepatic locations in AE patients are rare [35, 36] (note that our RDT can identify patients with pulmonary CE cysts although with lower reactivity [19]). In a previous study we found pulmonary echinococcosis in 4.9% of CE patients and none in AE patients [19]. Other possibilities for the positive test results may include serological cross-reactivity with related tapeworms or abortive *Echinococcus* infections. Therefore, it is necessary to carry out long-term follow-up examinations of individuals who show positive echinococcosis serology but have no cysts or lesions.

The main purpose of this study was to evaluate the performance of our ITC device under clinical field conditions. Therefore, individuals with clinical suspicion of echinococcosis were selected as study subjects. This could have resulted in some bias as the prevalence of echinococcosis in this subgroup was 34% (20.57% for CE and 13.45% for AE) which is far from the real prevalence in the population. For example, Li et al. reported a prevalence of 5.95% (1.94% for CE, 4.01% for AE and 0.00% for dual infection) in the Ganzi prefecture during the period 2001–2008 [20].

Human CE and AE are chronic diseases with a spectrum of clinical manifestations ranging from asymptomatic to serious, even life-threatening conditions [1]. Usually, most cases of echinococcosis in humans are diagnosed accidentally [8]. However, early diagnosis of the disease can greatly improve the management and treatment of patients. In order to accomplish this, an effective screening test is needed. Imaging methods are suitable for mass screening in underserved rural endemic areas, but they require well-trained staff and are time-consuming [37]. However, our RTD is a simple device that requires only 20 μl of whole blood [19]. The test takes less than 20 minutes and the result is displayed visually [19]. Moreover, with this device, testing can be carried out while patients are waiting and, therefore, the whole procedure is more convenient for patients than conventional immunological assays or imaging techniques. In conclusion, we suggest that the best method for early detection of *Echinococcus* infections would be preliminary mass screening programmes for at-risk populations in endemic areas using an RDT followed by confirmation of positive cases using imaging techniques. We think that our RDT fulfils the criteria for an echinococcosis screening test: the device is easy to use, quick and cheap, and has a high sensitivity, i.e. it essentially indicates suspicion of the disease that warrants confirmation. Importantly, our RDT can distinguish between both CE and AE that should help to guide subsequent diagnosis.

## Conclusions

In this study, we have shown that our previously developed RDT performed well under clinical field conditions and is a useful screening and complementary tool for the detection of echinococcosis in humans. Only patients with a positive result would need confirmation of their infection status by ultrasound imaging technique. However, it remains to be shown whether the RDT will perform equally well in mass screening studies (under real field conditions including all inhabitants of an endemic area).

## Abbreviations

AE: Alveolar echinococcosis
CE: Cystic echinococcosis
RDT: Rapid diagnostic test
